# Supplementary material for: Associations between Disc Hemorrhage and Primary Open-Angle Glaucoma Based on Genome-Wide Association and Mendelian Randomization Analyses
Source: Biomedicines. 2024 Oct 3;12(10):2253. doi: 10.3390/biomedicines12102253 (PMC11504051; doi:10.3390/biomedicines12102253)
Supplement: Supplementary file 1 [file biomedicines-12-02253-s001.zip › biomedicines-3206176-supplementary.pdf]

Supplementary Table S1. Instrumental variables for Mendelian Randomization for Disc Hemorrhage and Glaucoma

SNP	Chr	Position	Mapped Gene	Effect Allele	Other Allele	Effect Allel	Beta	SE	P-value	F
rs7589033	2	43649228	*THADA*	T	C	0.052	1.574	0.329	1.77E-06	310.49
rs9462784	6	42202149	*TRERF1*	C	T	0.111	1.329	0.286	3.31E-06	446.44
rs77127203	6	166267889	*PDE10A;LINC00473*	A	G	0.276	1.267	0.265	1.67E-06	824.80
rs80166622	10	5522787	*NET1;CALML5*	G	A	0.081	1.334	0.302	9.63E-06	340.43
rs80048012	10	80403228	*LINC00595;ZMIZ1-AS1*	T	G	0.053	1.471	0.327	6.77E-06	276.10
rs76143071	15	50723310	*USP8*	C	T	0.053	1.548	0.330	2.74E-06	305.94
rs113460962	17	4850748	*PFN1*	C	T	0.099	1.353	0.288	2.60E-06	418.02
rs11658281	17	8637070	*CCDC42*	T	G	0.135	1.393	0.282	7.78E-07	583.75
rs78583358	20	1840763	*LOC100289473;SIRPA*	T	G	0.095	1.308	0.294	8.50E-06	379.31
